# Inhibition of aldehyde dehydrogenase 1 enhances the cytotoxic effect of retinaldehyde on A549 cancer cells

**DOI:** 10.18632/oncotarget.19544

**Published:** 2017-07-25

**Authors:** Jin Won Park, Kyung-Ho Jung, Jin Hee Lee, Seung Hwan Moon, Young Seok Cho, Kyung-Han Lee

**Affiliations:** ^1^ Department of Nuclear Medicine, Samsung Medical Center, Seoul, Korea; ^2^ Samsung Advanced Institute for Health Sciences & Technology, Sungkyunkwan University School of Medicine, Seoul, Korea

**Keywords:** cancer, aldehyde dehydrogenase, retinaldehyde, cancer stem cell, ROS

## Abstract

We hypothesized that aldehyde dehydrogenase1 (ALDH1) protects cancer cells from retinaldehyde-induced cytotoxicity, and that targeting this enzyme would enhance the therapeutic effect of retinaldehyde. ALDEFLUOR™ assays showed high ALDH activity in A549 and H522 cancer cells and low activity in H1666 and T47D cancer cells. Immunoblots showed that expression of ALDH1A1 and ALDH1A3 was high in A549 and H522 cells, but low in H1666 cells. HPLC confirmed that N, N-diethylaminobenzaldehyde (DEAB) inhibits ALDH-mediated disposal of retinaldehyde in A549 cells and lysates. Treatment of A549 cells with retinaldehyde in the presence of DEAB augmented reactive oxygen species production and decreased glucose uptake and oxygen consumption. Importantly, DEAB substantially potentiated the ability of retinaldehyde to dose-dependently suppress the survival of A549 and H522 cells, whereas the added effect of DEAB was minor in H1666 and T47D cells. Gene silencing with specific siRNA revealed that ALDH1A1 contributed to protection of A549 cells against retinaldehyde toxicity. These results demonstrate that ALDH1 confers protection against retinaldehyde toxicity in cancer cells.

## INTRODUCTION

There is accumulating evidence that aldehyde dehydrogenase (ALDH) plays an important role in cancer [1–3]. ALDHs are a group of enzymes that catalyze the conversion of aldehydes to corresponding carboxylic acids [1]. This reaction can serve to protect cells against reactive aldehydes [4] that are potentially cytotoxic [5, 6]. Harmful effects of acetaldehyde range from increased reactive oxygen species (ROS) [7] to the formation of DNA and protein adducts [8]. Therefore, protection of cells from harmful aldehydes requires a rapid detoxification mechanism, which can be provided by their enzymatic disposal.

Among ALDHs, there is particularly great interest in ALDH1, which is widely used as a marker to identify cancer stem cells in malignancies including lung cancer [9]. However, ALDH1 is not merely a marker of cancer stemness, but also has important roles in tumor biology [10]. ALDH1 expression is associated with poor cancer prognosis [1, 2] and is involved in tumor drug resistance, oxidative stress, and differentiation. Therefore, a better understanding of the pathophysiologic function of ALDH1 in cancer cells is needed. The main substrate for ALDH1 is retinaldehyde [7, 10], which is obtained from food [11]. In the cell, retinaldehyde is oxidized to retinoic acid by ALDH1. Although previously considered active only on the retina, retinaldehyde is now known to modulate the biology of a diverse range of cell types [12, 13].

Lung cancers frequently exhibit intrinsic and acquired resistance to anticancer agents, including recent targeted therapies [14]. It is therefore beneficial to develop new strategies to treat lung cancer. A549 cells are human adenocarcinomic alveolar basal epithelial cells that were developed by culturing explanted lung cancer tissue of a male patient [15]. These cells are widely used as a preclinical research model to develop new treatments for lung cancer [16] and are known to express high levels of ADLH [17], making them an appropriate cell model for our study purpose.

In this study, we used A549 cells and other cancer cells to test the hypothesis that ALDH1 protects cancer cells from the cytotoxic effects of retinaldehyde and could therefore be a potential target for cancer therapy.

## RESULTS

### ALDH activity and major ALDH1 isoform expression

**Figure 1:**
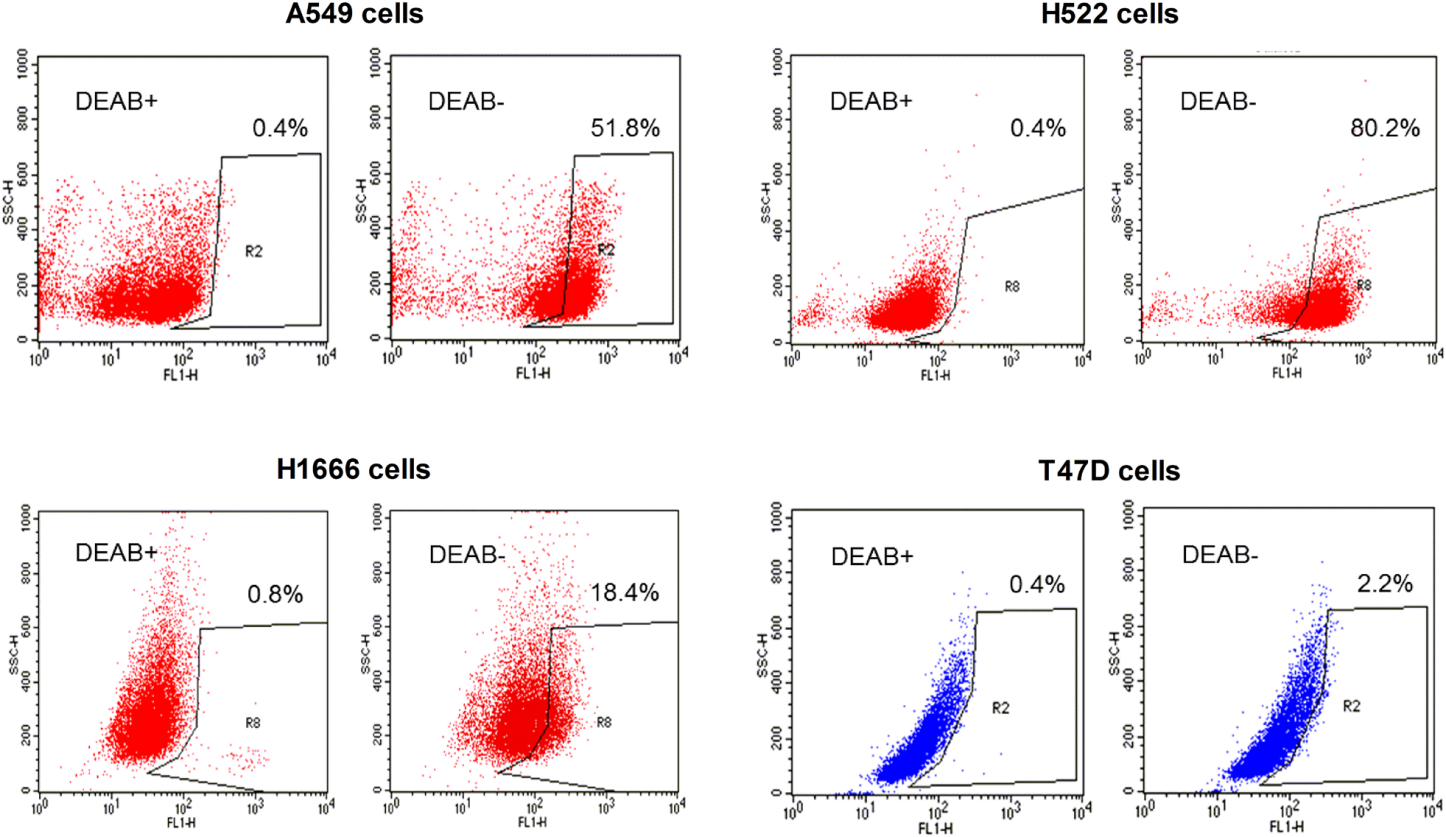
ALDEFLUOR™ assay measurements of ALDH activity Representative ALDEFLUOR™ assays with FACS analysis of A549, H522, and H1666 human lung cancer cells and T47D breast cancer cells. Cells incubated with ALDEFLUOR™ reagent green dye in the presence of DEAB (left) were used to establish baseline fluorescence to define the ALDEFLUOR™-positive area (R2). The ALDEFLUOR™-positive population was defined as cells showing a right-shift in fluorescence following incubation with ALDEFLUOR™ in the absence of DEAB (right).

We first evaluated the level of ALDH activity using ALDEFLUOR™ (BODIPY-aminoacetaldehyde) assays. The results of these assays showed that 51.8% of A549 cells and 80.2% of H522 cells were ALDH positive. In comparison, only 18.4% of H1666 cells and 2.2% of T47D cells were ALDH positive (Figure 1).

**Figure 2:**
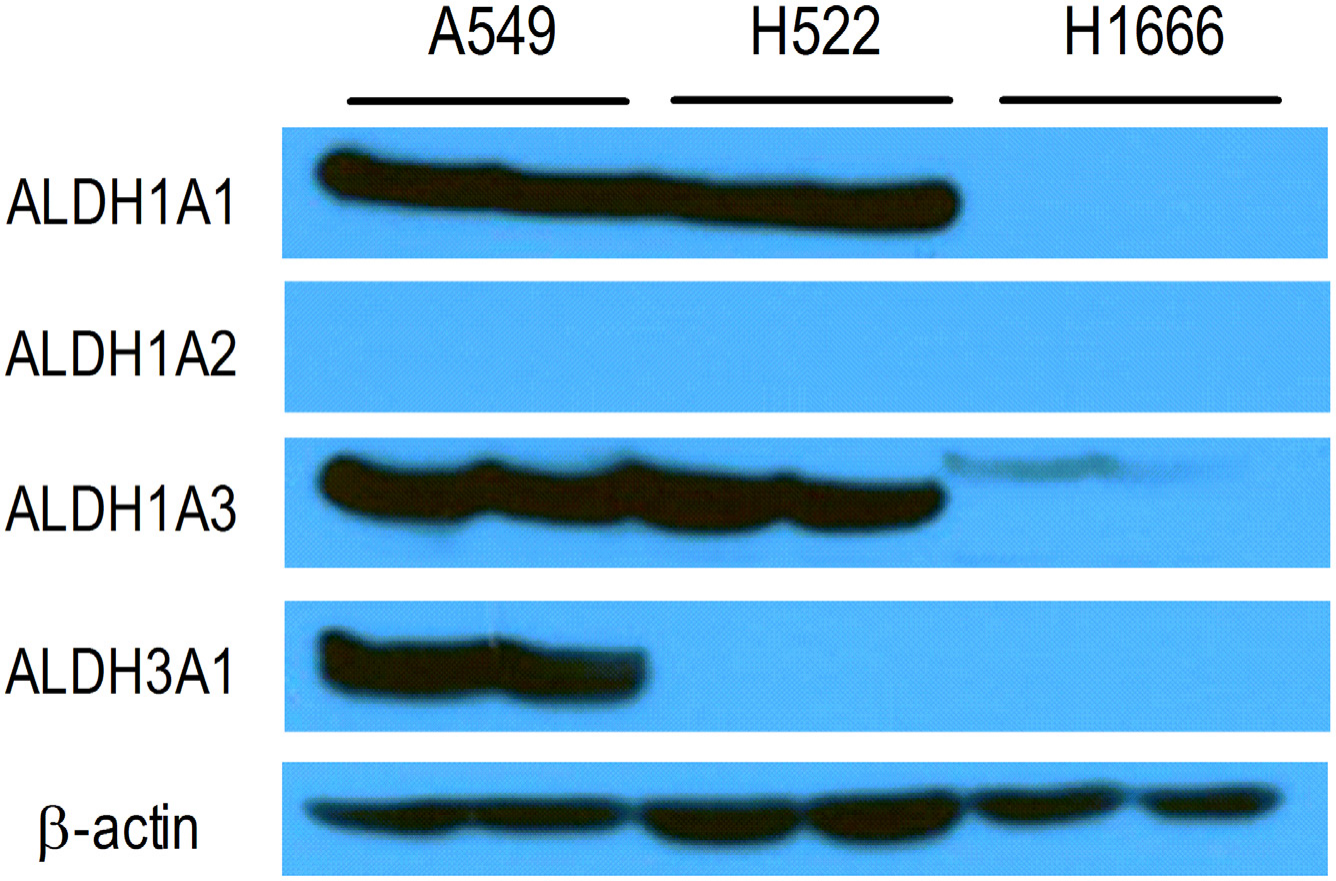
Immunoblots of lung cancer cell lines Immunoblot analysis of ALDH1A1, ALDH1A2, ALDH1A3, and ALDH3A1 in A549, H522, and H1666 human lung cancer cells. β-Actin was used as a loading control.

Immunoblotting demonstrated strong protein bands for ALDH1A1 and ALDH1A3 in A549 cells and H522 cells. A549 cells also showed strong protein bands for ALDH3A1 (Figure 2). H1666 cells showed faint protein bands only for ALDH1A3, and none of the cells tested showed detectable ALDH1A2 expression.

### A high level of retinaldehyde is maintained by ALDH inhibition

**Figure 3:**
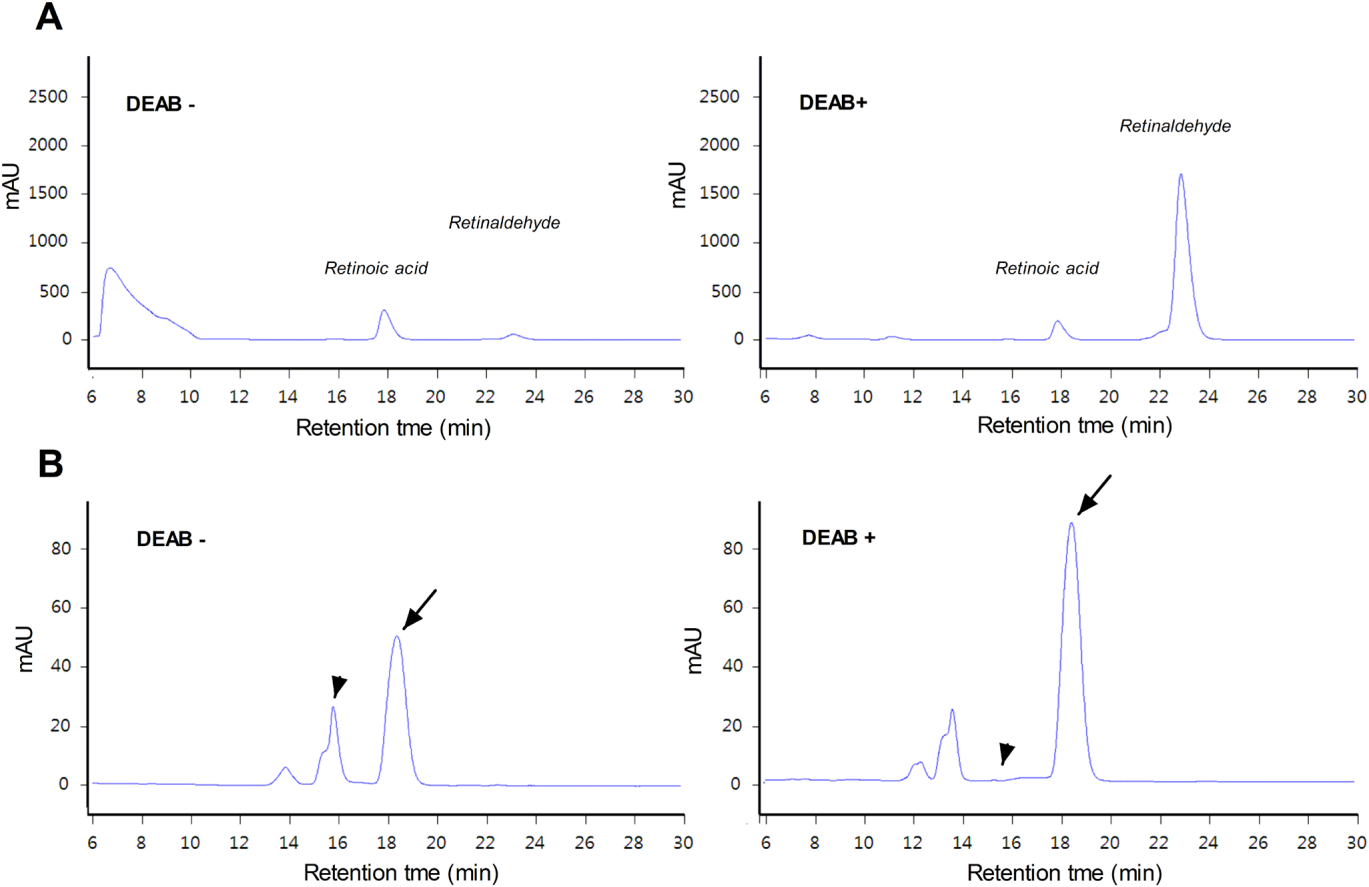
HPLC analysis of retinaldehyde and its metabolites **(A)** HPLC chromatograms of A549 cell lysates after 5-h incubation with retinaldehyde alone or a combination of retinaldehyde and DEAB. **(B)** HPLC chromatograms of cytosol samples prepared from A549 cells that had been treated for 1 h with retinaldehyde in the presence or absence of DEAB. Arrows indicate peaks corresponding to retinaldehyde. Arrowheads indicate the retention time for the peak attributed to retinoic acid that appeared in the absence of DEAB (left) but was no longer seen when DEAB was added (right).

High-performance liquid chromatography (HPLC) analysis of all-trans retinaldehyde, retinoic acid, retinol, and N, N-diethylaminobenzaldehyde (DEAB) standards showed retention times of approximately 25 min, 18 min, 19 min, and 5 min, respectively (Supplementary Figure 1). When retinaldehyde was incubated with A549 cell lysate for 5 h, a very small retinaldehyde peak and a larger retinoic acid peak were observed. When we repeated the experiment in the presence of DEAB, a commonly used selective inhibitor of ALDH, the retinaldehyde peak was greatly increased, while the retinoic acid peak became smaller (Figure 3A). We also performed HPLC of cell lysates after A549 cells had been incubated with retinaldehyde for 1 h in the absence or presence of DEAB. Under this condition, retinaldehyde and retinoic acid peaks appeared at somewhat earlier retention times, which was thought to represent trans-cis isomerization in living cells (Figure 3B). DEAB caused a substantial enlargement of the retinaldehyde peak, while the retinoic acid peak disappeared (Figure 3B). These findings demonstrate that ALDH can rapidly oxidize retinaldehyde to retinoic acid, but a high level of retinaldehyde is maintained when ADLH activity is blocked by DEAB.

### Retinaldehyde combined with ALDH inhibition increases reactive oxygen species in A549 cells

**Figure 4:**
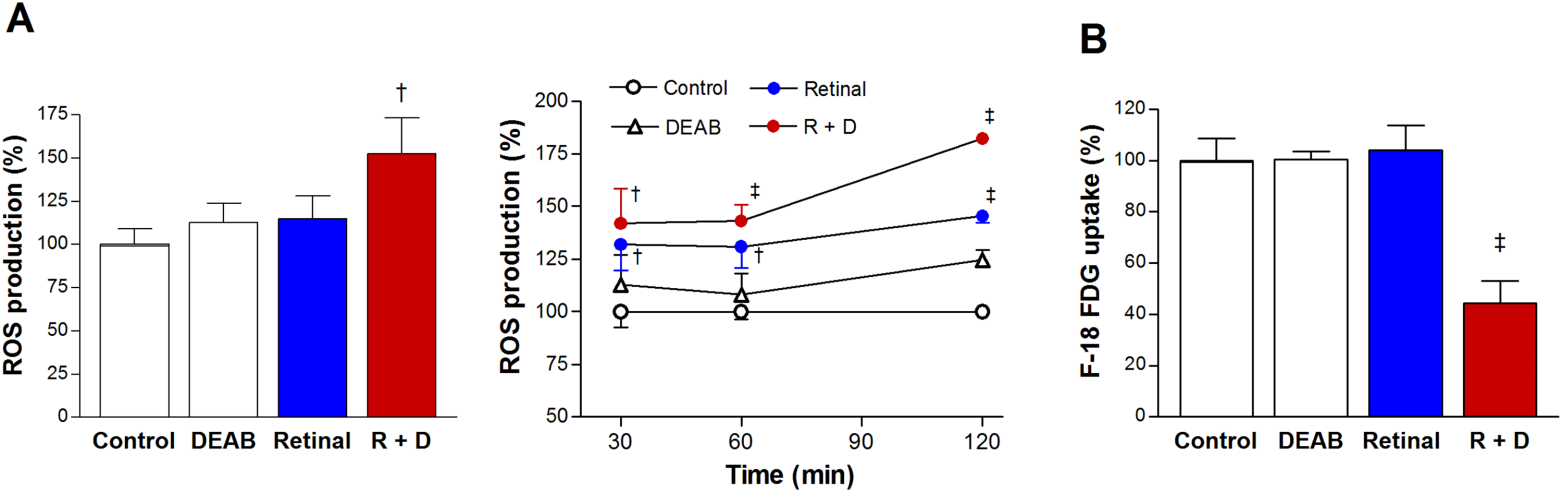
Retinaldehyde with ALDH inhibition stimulates ROS production and reduces FDG uptake **(A)** Intracellular ROS was measured by CMH2DCFDA fluorescence in A549 cells treated for 2 h with 30 μM retinaldehyde and/or 100 μM DEAB (left). The temporal course of ROS production from 30 min to 2 h of treatment as above (right). **(B)** FDG uptake of A549 cells after 2 h treatment as above. All data are mean ± SD of percentage values relative to untreated controls (n = 5 for A, and n = 4 for B) obtained from a single representative experiment. †*P* <0.005, ‡*P* <0.001, compared with controls treated with DMSO vehicle.

In initial experiments performed at a single time point of 2 h, a combination of retinaldehyde and DEAB increased ROS production in A549 cells to 152.5 ± 20.6% of that in controls, whereas the stimulatory effect of retinaldehyde alone did not reach statistical significance (Figure 4A). More careful examination of time-course effects showed that ROS stimulation by combined retinaldehyde and DEAB occurred rapidly, and that retinaldehyde alone also had a mild stimulatory effect. Levels of ROS stimulated by combined retinaldehyde and DEAB reached 141.9 ± 16.6% of control levels by 30 min and further increased to 182.2 ± 1.3% by 2 h (Figure 4A). Retinaldehyde alone increased ROS to 145.5 ± 3.1% of control levels by 2 h (Figure 4A), which was significantly lower than that achieved in the presence of additional DEAB (*P* <0.001).

### Retinaldehyde combined with DEAB suppresses glucose uptake and oxygen consumption

When used alone, neither retinaldehyde nor DEAB significantly influenced FDG uptake of A549 cells. However, in the presence of DEAB, retinaldehyde severely reduced cellular FDG uptake to 44.4 ± 8.7% of controls (Figure 4B).

**Figure 5:**
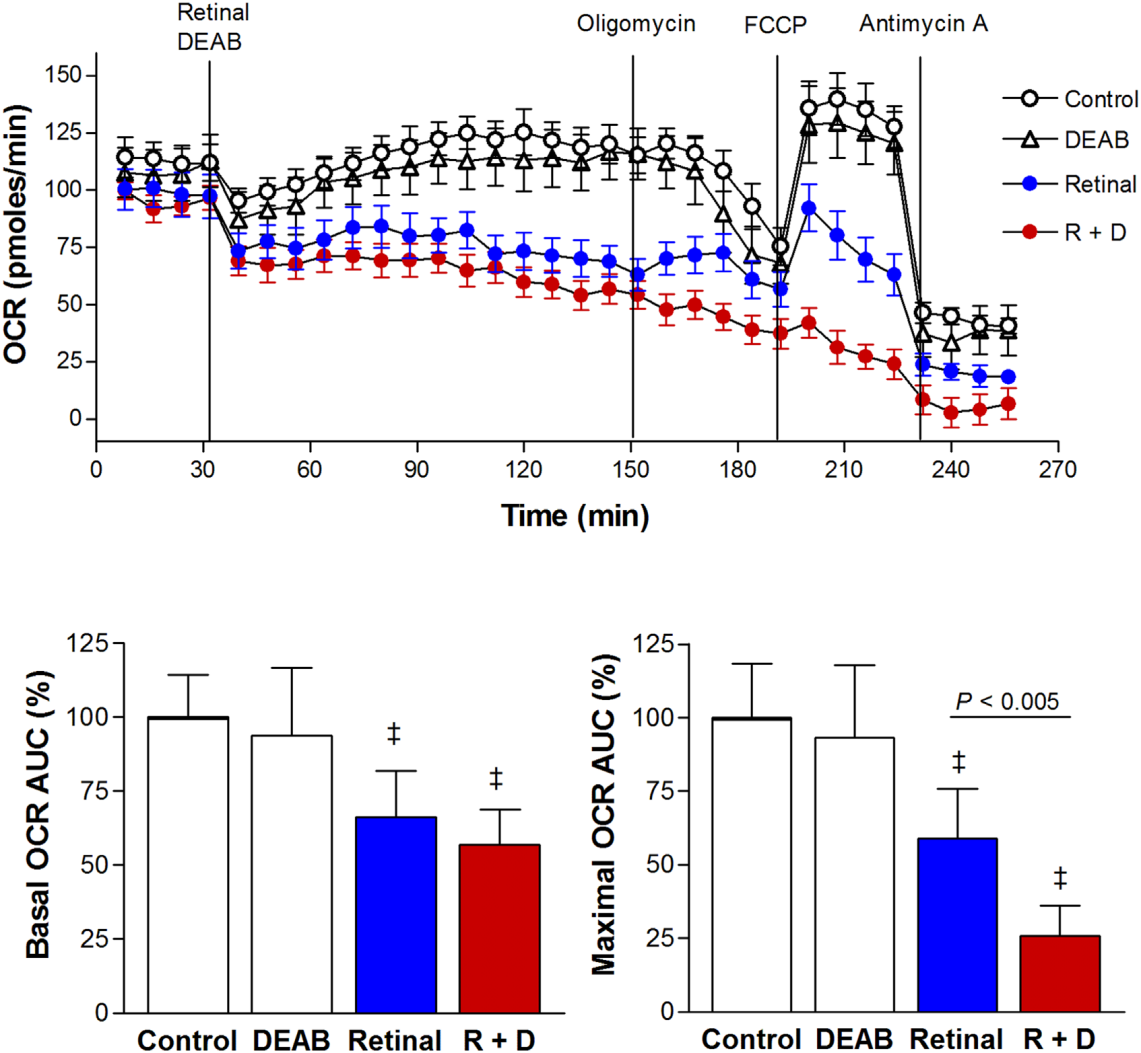
ALDH inhibition potentiates the ability of retinaldehyde to suppress cellular OCR OCR of A549 cells treated as above at baseline (30 to 150 min) and following sequential addition of oligomycin, FCCP, and antimycin A (top). AUC of the measured OCR of each group of cells at baseline (bottom, left) and during FCCP stimulation (bottom, right). Data are mean ± SD of percentage values relative to untreated controls obtained from a single representative experiment with 5 samples per group. ‡*P* <0.001, compared with controls.

We therefore investigated how retinaldehyde influences the oxygen consumption rate (OCR) of A549 cells. Used alone, retinaldehyde gradually caused a moderate reduction of basal OCR. The area under the curve (AUC) over 2 h of treatment reached 66.1 ± 15.6% of control level. Co-treatment with retinaldehyde and DEAB decreased the 2 h AUC of OCR to 56.7 ± 12.1% of controls (Figure 5). When the capacity for maximal OCR was evaluated with the proton gradient uncoupler carbonylcyanide-p-trifluoromethoxyphenylhydrazone (FCCP), combined treatment with retinaldehyde and DEAB showed a prominently greater effect. Thus, during FCCP treatment, the AUC of OCR was modestly reduced to 59.0 ± 16.8% of controls by retinaldehyde alone, but was markedly suppressed to 25.7 ± 10.5% of controls by addition of DEAB (Figure 5).

### Potentiation of retinaldehyde cytotoxicity by DEAB

**Figure 6:**
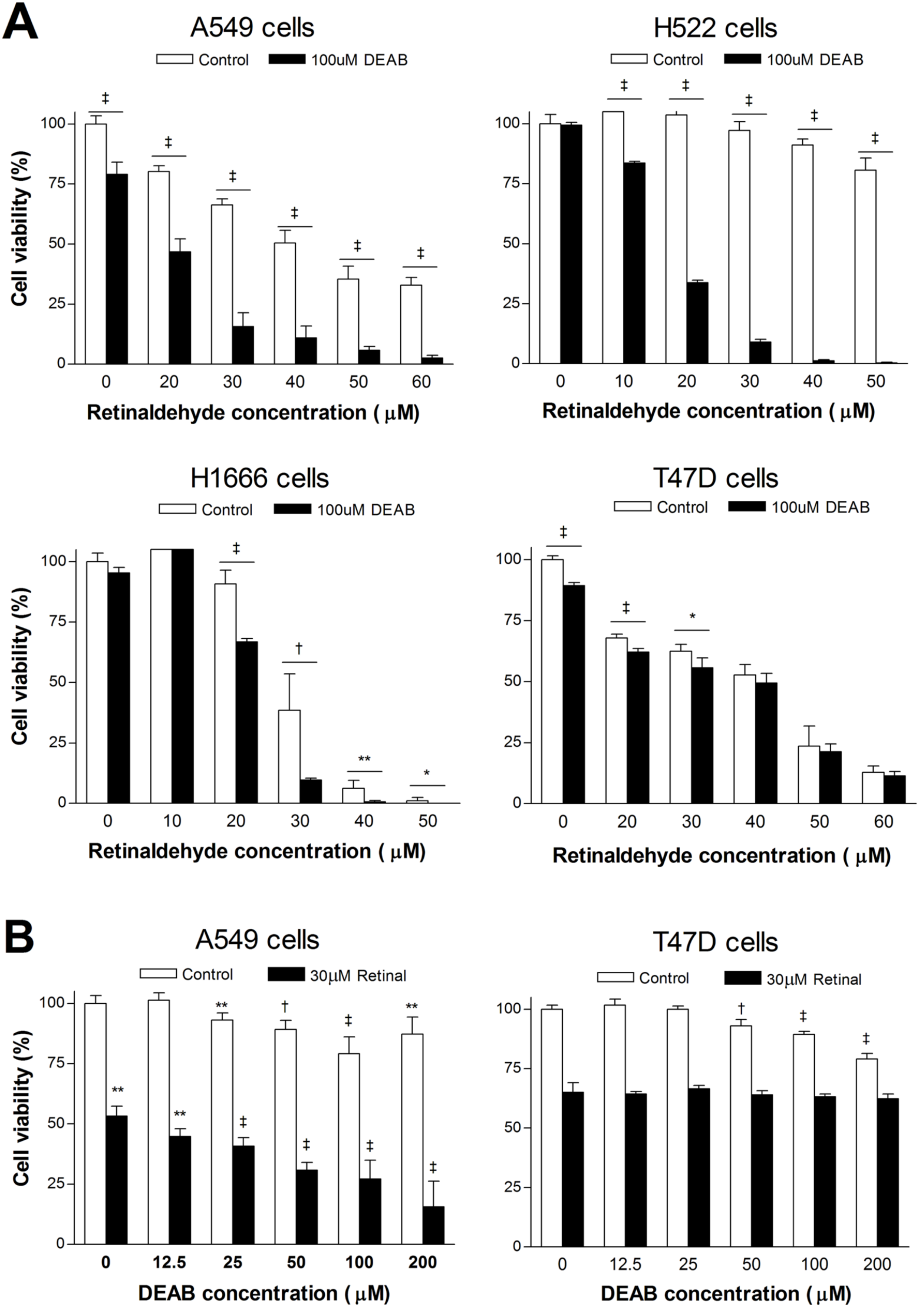
Effects of retinaldehyde and ALDH inhibition on cell viability **(A)** SRB assays of A549, H522, H1666, and T74D cell survival after 24 h treatment with graded doses of retinaldehyde with or without 100 μM DEAB. **(B)** SRB assays of A549 and T74D cell survival after 24 h treatment with graded doses of DEAB with or without 30 μM retinaldehyde. Bars are mean ± SD of percentage values relative to untreated controls (n = 5) from a single representative experiment. ^*^P <0.05, ^**^P <0.01, ^†^*P* <0.005, ^‡^*P* <0.001, compared with untreated controls.

Sulforhodamine-B (SRB) assays showed that a high concentration of retinaldehyde alone decreased viability of all cancer cells tested, but with different magnitudes. When treated with 50 μM retinaldehyde, cell viability was lowest for H1666 cells (1.1 ± 1.4%; *P* <0.001 compared with all other cell types), followed by T47D cells (23.7 ± 8.3%) and A549 cells (35.4 ± 5.4%), and was highest for H522 cells (80.7 ± 5.1%; *P* <0.001 compared with all other cell types; Figure 6A). The potentiating effect of combined treatment with DEAB on retinaldehyde cytotoxicity varied according to cell type. Although DEAB did not further reduce the viability of T47D cells, it significantly reduced viability of other cell types and had a particularly marked effect on H522 cells. Thus, in the presence of DEAB, 20 μM retinaldehyde reduced A549 cell viability from 80.2 ± 2.4% to 46.8 ± 5.4%, H1666 cell viability from 90.8 ± 5.6% to 66.8 ± 3.1%, and H522 cell viability from 103.6 ± 2.0% to 33.7 ± 2.4% (all *P* <0.001; Figure 6A). The magnitude of reduction in viability was significantly greater for H522 cells compared with all other cell types (*P* <0.001).

Graded doses of DEAB showed only weak effects on A549 and T47D cell viability (Figure 6B). When 30 μM of retinaldehyde was added, A549 cell viability decreased in a DEAB concentration-dependent manner, reaching 15.6 ± 10.5% of control levels with 200 μM DEAB (Figure 6B). In contrast, T47D cell viability in response to graded doses of DEAB was not influenced by the presence of 30 μM retinaldehyde (Figure 6B).

### Gene silencing shows that ALDH1A1 contributes to protection against retinaldehyde

**Figure 7:**
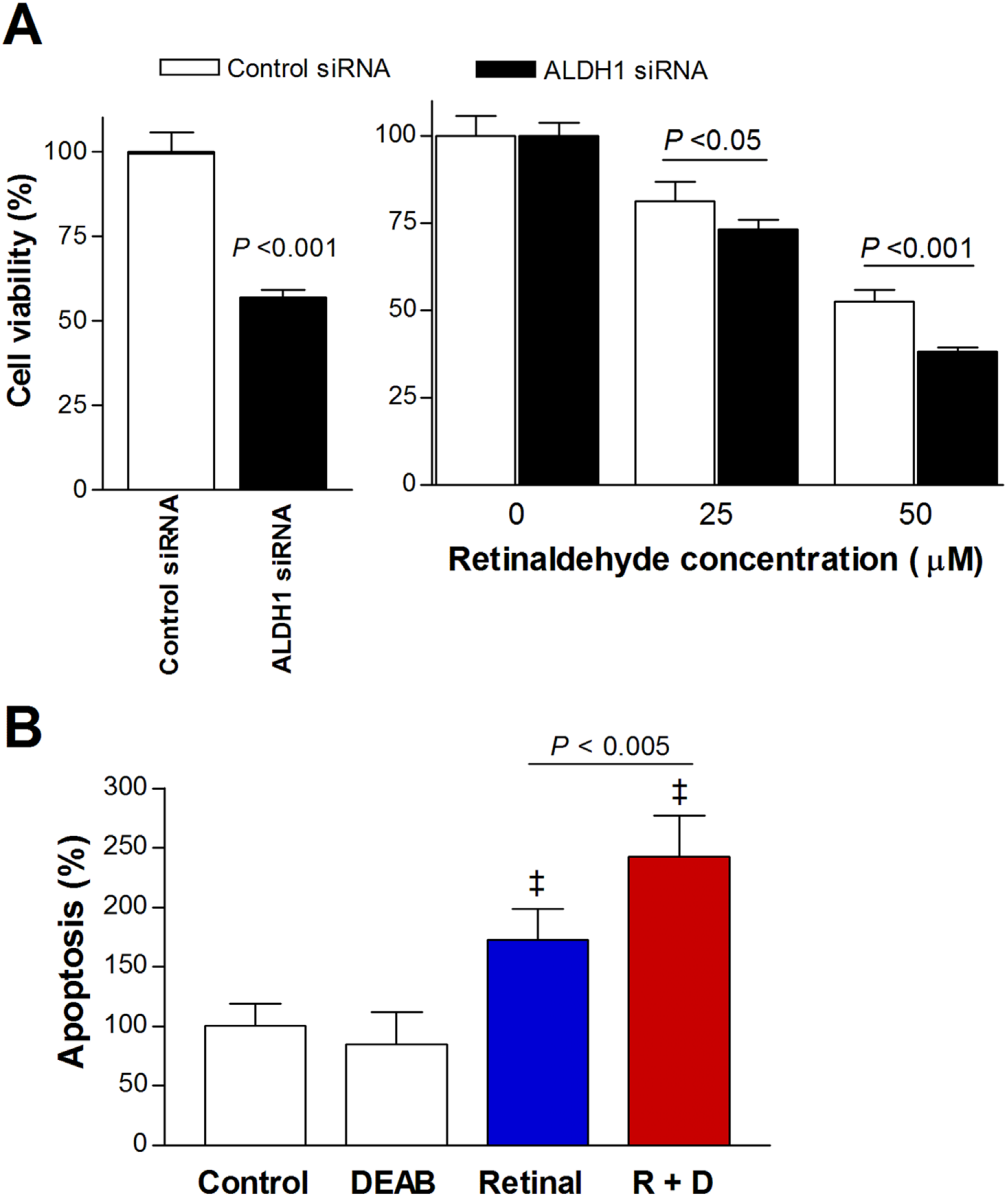
Retinaldehyde cytotoxicity under ALDH1A1 silencing and apoptosis induced by retinaldehyde **(A)** SRB assays of A549 cells 48 h after transfection with ALDH1A1-specific and scrambled control siRNA (left). Relative viability of transfected A549 cells is shown after 24-h treatment with 25 and 50 μM retinaldehyde (right). **(B)** Immunosorbent assays of cell death enzymes showing influence of 15-h treatment with retinaldehyde and/or DEAB on A549 cell apoptosis. All bars are mean ± SD of percentage values relative to controls obtained from a single representative experiment (n = 4, A; n = 6, B). ‡*P* <0.001, compared with untreated control cells.

At 2 days following transfection with ALDH1A1-specific siRNA, A549 cell viability was 57.0 ± 2.2% of that of control cells transfected with scrambled siRNA (Figure 7A). This was thought to represent the slower proliferation of cells with ALDH1A1 silencing. In cells transfected with control siRNA, treatment with 50 μM retinaldehyde reduced cell viability to 52.6 ± 3.3% of untreated cells. In cells transfected with ALDH1A1 siRNA, 50 μM retinaldehyde caused a significantly greater reduction in viability, which reached 38.2 ± 1.1% of that of untreated cells (Figure 7A).

### Retinaldehyde combined with DEAB increases apoptotic cell death

Cell death enzyme-linked immunosorbent assay (ELISA) showed that apoptosis in A549 cells treated with retinaldehyde alone increased to 172.5 ± 26.2% of that in untreated control cells. Treatment with a combination of retinaldehyde and DEAB resulted in a significantly greater rate of apoptosis that reached 242.1 ± 35.0% of control levels (Figure 7B).

## DISCUSSION

Our study demonstrates that retinaldehyde efficiently and dose-dependently suppresses cancer cell survival. In A549 cancer cells, retinaldehyde toxicity was substantially potentiated by a specific ALDH inhibitor. This enhanced cytotoxic effect was accompanied by stimulation of ROS production, impairment of mitochondrial respiration, and increased apoptotic cell death.

In our study, we used A549 human cancer cells because they are widely used as an *in vitro* model to develop new lung cancer treatments and are known to have high ALDH activity [17]. In comparison, T47D human breast cancer cells have low ALDH activity [2]. We also used additional human adenocarcinoma cell lines, H522 cells and H1666 cells, which we found to have high and low ALDH activity, respectively. ALDEFLUOR™ assay results are largely determined by ALDH1 activity (and particularly ALDH1A1 activity), although ALDH3A1 and ALDH7A1 can also contribute [10]. In addition to ALDEFLUOR™ assays, we also performed immunoblots and found that A549 cells and H522 cells have strong ALDH1A1 and ALDH1A3 expression but no detectable ALDH1A2 expression. H1666 cells had weak ALDH1A3 expression with undetectable expression of ALDH1A1. Our results for A549 cells are consistent with a previous study showing ALDH1A1 and ALDH3A1 expression by these cells [17].

Retinaldehyde is rapidly metabolized either by oxidation to retinoic acid via ALDHs or by reduction to retinol via retinol dehydrogenases [18]. In a previous study, retinaldehyde was rapidly metabolized by retinal epithelial cells and became undetectable by 4.5 h, leaving retinol, retinoic acid, and retinyl esters [19]. Under our HPLC conditions, all-trans retinol and retinoic acid were identified as distinct peaks that eluted earlier than the retinaldehyde peak. When retinaldehyde was applied to A549 cell lysate or to live A549 cells, there was a rapid decline in retinaldehyde concentration with an increase in its metabolites. However, a high retinaldehyde concentration was maintained in the presence of DEAB. This demonstrates that blocking ALDH activity delays enzymatic removal of retinaldehyde in cells. A shift in the retention times of retinaldehyde and retinoic acid after incubation with cells might represent trans-cis isomerization of retinoids. All-trans retinaldehyde can be converted into isoforms with different HPLC retention times [20], and cis isoform isomerization of all-trans retinaldehyde has been reported in retinal epithelial cells [21].

Reactive aldehydes can increase ROS production. This is exemplified by the reported ability of acetaldehyde to stimulate ROS in hepatic stellate cells [22, 23] and neuroblastoma cells [24]. Retinaldehyde has been shown to similarly stimulate ROS generation in retinal epithelial cells [25], and this response has been proposed to contribute to retinal macular degeneration [26]. A recent study found that patient tissue-derived ovarian cancer cells with strong ALDH activity had lower ROS levels [27]. In our study, ROS levels were increased when A549 cells were treated with retinaldehyde in the presence of DEAB. Taken together, these findings are consistent with the notion that ALDH mitigates cellular oxidative stress by scavenging aldehydes [7].

When we evaluated mitochondrial function, glucose uptake by A549 cells was unaffected by retinaldehyde or DEAB alone, but was significantly suppressed by a combination of retinaldehyde and DEAB. Also, oxygen consumption rate was moderately decreased by retinaldehyde alone and appeared to be suppressed to a slightly greater extent when DEAB was present. Furthermore, a profound difference was observed for FCCP-stimulated OCR, which represents maximal mitochondrial respiratory capacity. Cells treated with a combination of retinaldehyde and DEAB displayed a nearly complete flat response to FCCP, whereas the response was only modestly reduced by retinaldehyde alone. These findings demonstrate that cancer cell mitochondrial function is suppressed by retinaldehyde in the absence of ALDH activity.

A suppressive effect of retinaldehyde on cell survival has been previously demonstrated in retinal epithelial cells [25]. When we examined cancer cell viability by SRB assays, retinaldehyde alone dose-dependently decreased the viability of all cancer cells tested but with different potencies. The cytotoxic effect was greatest for H1666 cells, followed by T47D cells and A549 cells, whereas it was minimal for H522 cells. Conversely, DEAB-induced susceptibility to retinaldehyde was greatest for H522 cells and weakest for H1666 cells and T47D cells. These findings suggest that ALDH offers protection against retinaldehyde toxicity in a manner that is effectively blocked by DEAB.

A potential association between cytotoxicity of retinoids and the activity of enzymes involved in their metabolism has previously been pointed out. In kidney HEK293 cells, retinaldehyde toxicity was decreased by expression of retinol dehydrogenase [28]. In Sertoli cells, ALDH inhibition potentiated retinol-induced ROS production and cell damage [29]. In another study, forced expression of retinol dehydrogenase in MCF7 breast cancer cells lacking ALDH resulted in increased cytotoxicity of retinol that was attributed to the formation of retinaldehyde [30].

DEAB is widely used in ALDEFLUOR™ assays to identify stem cells that express ALDH1. Although generally considered a selective ALDH1A1 inhibitor, DEAB is also able to block ALDH2, an isoform responsible for alcohol-induced acetaldehyde detoxification [31]. Furthermore, DEAB has also been reported to inhibit other ALDH1 isoforms including ALDH1A2, ALDH1A3, and ALDH1B1 [32]. Therefore, the ability of DEAB to potentiate retinaldehyde toxicity cannot be attributed to blockage of the activity of a specific ALDH isoform.

Using gene silencing experiments, we confirmed that retinaldehyde cytotoxicity was significantly potentiated when A549 cells were transfected with siRNA specific for ALDH1A1 compared with scrambled siRNA. Our focus on silencing ALDH1A1 was motivated not only because it was abundantly expressed in A549 cells, but also because of its important roles in cancer biology. ALDH1A1 is one of three highly conserved cytosolic isozymes that catalyze the oxidation of retinaldehyde to retinoic acid with high affinity. In cancer, this isoform has an important role in modulating cell proliferation and differentiation. ALDH1A1 overexpression, which is observed in various cancer types, has been associated with cancer cell stemness, treatment resistance, and poor patient prognosis [1]. Our results indicate that ALDH1A1 contributes to a protective effect against retinaldehyde toxicity that is blocked by DEAB. Nevertheless, it should be mentioned that we did not evaluate the effect of silencing other ALDH1 isoforms, and it is therefore possible that ALDH1A3 also contributes to protection against retinaldehyde cytotoxicity.

Finally, cell death ELISA demonstrated that the cytotoxic effect of retinaldehyde was accompanied by increased apoptosis. A previous study showed that retinaldehyde induced retinal epithelial cell death by triggering apoptosis, and that this was attributed to ROS-induced DNA damage and p53 activation [25]. Furthermore, SH-SY5Y neuroblastoma cancer cells treated with acetaldehyde showed cytotoxic effects via apoptotic signaling and inhibition of cell survival pathways [24]. Our results suggest that retinaldehyde cytotoxicity in cancer cells with blocked ALDH activity might also occur through promotion of apoptosis.

Our findings support the possibility that combining retinaldehyde with drugs that inhibit ALDH1A1 might offer potential benefit for cancer treatment. Retinaldehyde is normally present in living bodies, and cancer stem cells use significant amounts of retinaldehyde for differentiation [1, 2]. This suggests that ALDH inhibitors might be useful for cancer treatment without the need for additional retinaldehyde. It should be pointed out, however, that DEAB has limited specificity for ALDH isoforms, and newer ALDH inhibitors with greater specificity might be required for this purpose.

In conclusion, our results confirm that retinaldehyde exerts dose-dependent cytotoxicity on cancer cells, and that this effect is potentiated by blocking ALDH1 activity. These data indicate that an important role of ALDH1 in cancer cells is to confer protection by detoxifying retinaldehyde through enzymatic disposal, which could offer a potential therapeutic target.

## MATERIALS AND METHODS

### Cell culture

A549, H522, and H1666 human lung adenocarcinoma cells and T47D human breast cancer cells were from the Korea Cell Line Bank (Republic of Korea, Seoul) and the American Type Culture Collection (Manassas, VA). Cells were maintained in RPMI 1640 media (Lonza, Switzerland) supplemented with 10% fetal bovine serum (Serena, Germany) and 1% penicillin/streptomycin (Lonza) at 37°C and 5% CO_2_ in a humidified atmosphere. Media were replaced with phenol red-free RPMI 1640 (Gibco, MA) with 10% fetal bovine serum 48 h before experiments.

### ALDH activity assay

ALDH activity was measured using an ALDEFLUOR™ assay kit (Stemcell Tech, BC, Canada) according to the manufacturer’s manual. Cells were resuspended in ALDEFLUOR™ assay buffer containing 1 μM ALDEFLUOR™ dye. ALDH activity was blocked with 15 μM of DEAB, a commonly used selective inhibitor of ALDH isoenzymes in cancer and stem cells. After incubation at 37°C and 5% CO_2_ for 30 min in a humidified atmosphere, cells were washed with ALDEFLUOR™ assay buffer. Finally, 10,000 cells per sample were analyzed using a FACS Calibur flow cytometer with CellQuest software (Becton-Dickinson, NJ).

### Immunoblotting

Cells were washed with PBS and solubilized in PRO-PREPTM protein extraction solution (iNtRON biotechnology, Korea) for 15 min at 4°C. Cell debris was eliminated by centrifugation at 14,000 rpm for 10 min at 4°C. The supernatant was analyzed for protein content by the Bradford method, and 20 μg of protein was separated on a 10% polyacrylamide gel. The protein was transferred to a hydrobond ECL nitrocellulose membrane (Amersham Biosciences; Piscataway, NJ) and incubated overnight at 4°C with polyclonal antibody against ALDH1A1 (Abcam; Cambridge, MA; 1:1000 dilution), ALDH1A2 (Abcam; Cambridge, MA; 1:1000 dilution), ALDH1A3 (Genetex; Cambridge, MA; 1:1000 dilution), or ALDH3A1 (Cusabio; CA; 1:1000 dilution) in Tris-buffered saline (50 mM Tris, pH 7.5, 150 mM NaCl) with 0.05% polysorbate-20 containing 5% skim milk. After washing three times for 10 min with Tris-buffered saline with Tween 20, the membrane was incubated with secondary antibodies for 1 h at room temperature. Immune reactive protein was detected with an enhanced chemiluminescence kit and quantified using Quantity One software (Bio-Rad Laboratories, CA).

### HPLC analysis

HPLC analysis was performed with two types of sample. The first was untreated A549 cell lysates that were reacted with 100 μg retinaldehyde with or without 100 μg DEAB at 37°C for 5 h. HPLC was performed on a Spectra system P4000 (Thermo, MA) equipped with a reverse-phase C18 column (4.6 ^*^ 250 mm) (YMC, Japan) and a UV2000 detector. Elution was performed with 80% acetonitrile in water for 30 min at a flow rate of 1 ml/min. Samples were analyzed at specific absorption wavelength of 298 nm.

The second type of sample was obtained from A549 cells that had been treated with 30 μM retinaldehyde with or without 100 μM DEAB for 1 h. Cells were washed with phosphate-buffered saline and lysed by pumping through a 23-G needle in 100 μl distilled water. After centrifugation at 14,000 rpm for 10 min, the supernatants were subjected to HPLC analysis.

### ROS production

Cells were cultured in 96-well black plates. Six hours before measurement, the culture medium was replaced with phenol red-free RPMI 1640 containing 10% fetal bovine serum and 10 μM CMH2DCFDA (Molecular Probes, Invitrogen, CA). Fluorescence was measured on a microplate reader using 490 nm excitation and 510 to 570 nm emission wavelengths.

### FDG uptake

Cells were incubated with 175-370 kBq FDG for 40 min at 37°C and then rapidly washed twice with cold phosphate-buffered saline, lysed with 0.01 N NaOH, and measured for cell-associated radioactivity on a high-energy γ counter (PerkinElmer, MA).

### OCR

Cells were seeded into Seahorse XF24 24-well plates (Seahorse Bioscience, MA) at 20,000 cells per well and equilibrated with 525 μl phenol red-free RPMI 1640 (pH 7.4) without sodium bicarbonate or fetal bovine serum at 37°C without CO_2_ for 1 h before the assay. Oxygen concentration of the media was measured on a Seahorse XF24 extracellular flux analyzer (Seahorse Bioscience, MA) with solid state sensor probes. OCR was measured during basal respiration and after treatment with retinaldehyde with or without DEAB. Oligomycin (4 μM, Sigma) was used to inhibit complex V, FCCP (10 μM, Sigma) was used to uncouple the proton gradient, and antimycin A (10 μM, Sigma) was used to inhibit complex III. These agents were sequentially added to the media of cells, while OCR was automatically calculated, recorded, and plotted using Seahorse XF24 software version 1.8. Mean OCR over certain time points was quantified as the AUC.

### ALDH1A1 knockdown by siRNA

Cells were transfected with ALDH1A1 siRNA (Santa Cruz Biotechnology, TX) or a negative control siRNA (Cell Signaling Technology, MA) in Opti-MEM with lipofectamine RNAi-MAX (Invitrogen, CA) according to the manufacturer’s manual. For transfection, 200 nM of siRNA was diluted in 25 μl Opti-MEM transfection medium (solution A). Solution B was prepared by adding 1.5 μl RNAimax transfection reagent to 25 μl Opti-MEM transfection medium. Solutions A and B were mixed and incubated for 5 min at room temperature. This mixture was added to 96-well plates and incubated in a CO_2_ incubator at 37°C. Cells were further maintained for 48 h before drug treatment.

### Cell viability assay

Cells were fixed with 10% (w/v) trichloroacetic acid and stained with SRB for 30 min. Excess dye was removed by repeated washing with 1% (v/v) acetic acid, and protein-bound dye was dissolved in 10 mM Tris base solution for determination of optical density at 510 nm on a micro-plate reader.

### Apoptosis assay

Apoptosis was evaluated using a cell death detection ELISA plus kit (Roche, Switzerland). Briefly, after cell treatment for 15 h, culture media was replaced with fresh media, and cells were harvested with lysis buffer 1 h later. Cell lysates were incubated in streptavidin-coated plates with 80 μl of a mixture anti-histone-biotin and anti-DNA-POD with shaking at RT for 2 h. After washing with incubation buffer, the plates were reacted with 2,2'-azinobis (3-ethylbenzothiazoline-6-sulfonic acid)-diammonium salt (ABTS) solution 10-20 min until the color of the buffer changed. Samples were finally analyzed at 405 nm on a micro-plate reader.

### Data analysis

Data are expressed as the mean ± SD. Significance of difference was analyzed by two-tailed unpaired Student’s t-tests for two groups and by ANOVA with Tukey post-hoc tests for three or more groups. *P* values <0.05 were considered statistically significant.

## SUPPLEMENTARY MATERIALS FIGURE


