# Supplementary material for: Inhibition of aldehyde dehydrogenase 1 enhances the cytotoxic effect of retinaldehyde on A549 cancer cells
Source: Oncotarget. 2017 Jul 25;8(59):99382–93. doi: 10.18632/oncotarget.19544 (PMC5725100; doi:10.18632/oncotarget.19544)
Supplement: Supplementary file 1 [file oncotarget-08-99382-s001.pdf]

Inhibition of aldehyde dehydrogenase 1 enhances the cytotoxic effect of retinaldehyde on A549 cancer cells

SUPPLEMENTARY MATERIALS

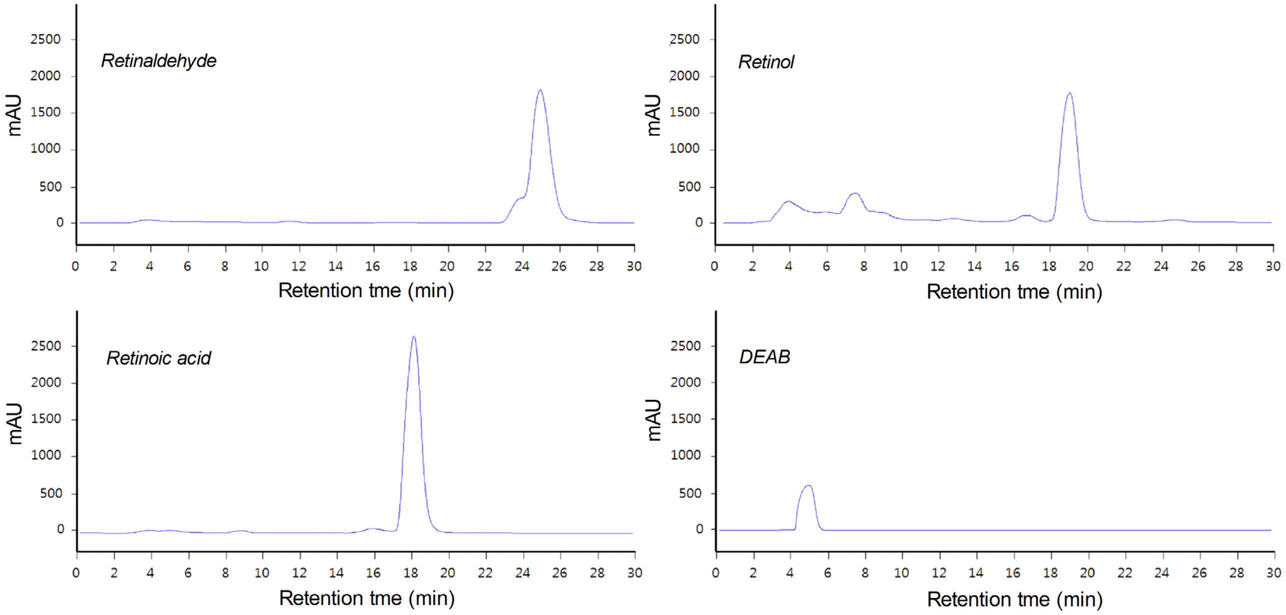

Supplementary Figure 1: Retention times of retinaldehyde and metabolite standards on HPLC. HPLC traces of retention peaks for 100 µg of all-trans retinaldehyde, retinol, retinoic acid, and DEAB standards are shown.
